# Supplementary material for: Causal Relationship between Aging and Anorexia Nervosa: A White-Matter-Microstructure-Mediated Mendelian Randomization Analysis
Source: Biomedicines. 2024 Aug 16;12(8):1874. doi: 10.3390/biomedicines12081874 (PMC11351342; doi:10.3390/biomedicines12081874)
Supplement: Supplementary file 1 [file biomedicines-12-01874-s001.zip › Supplementary Figure/Figure S4/Singe SNP plot of MR analysis for TL to ubm-b-1697.pdf]

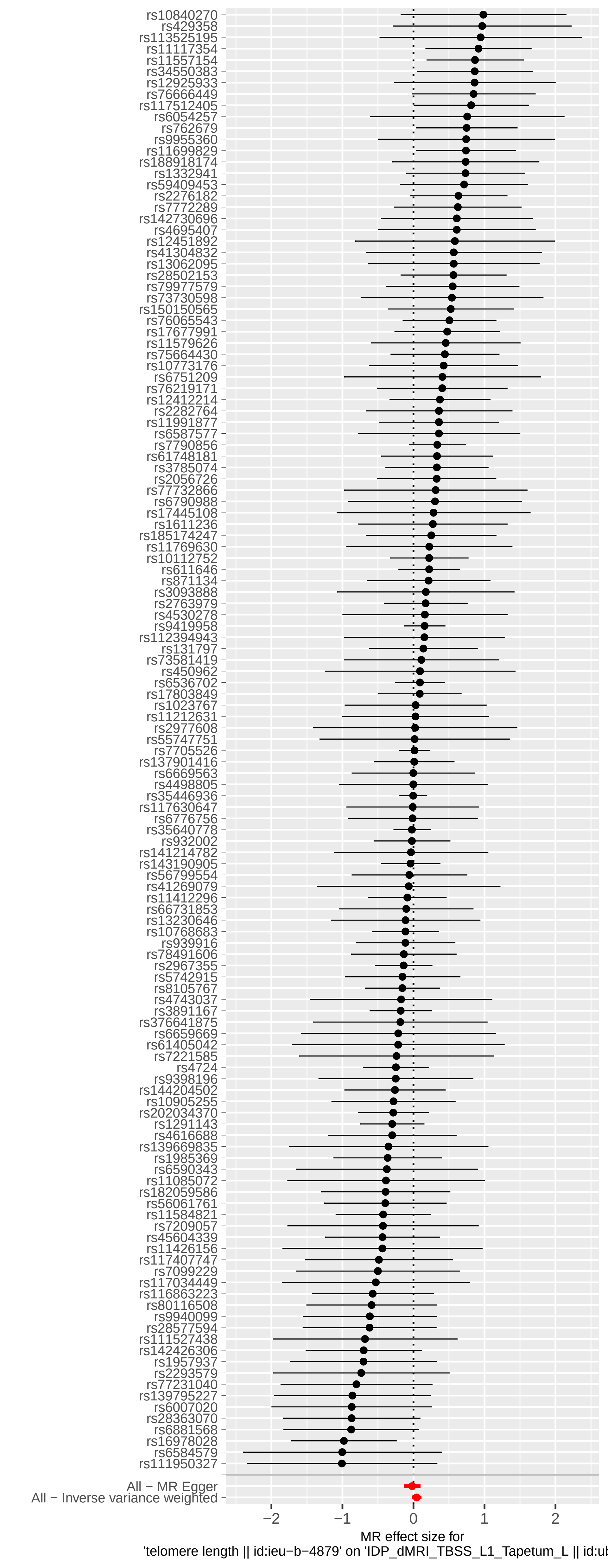

rs10840270
rs429358
rs113525195
rs11117354
rs11557154
rs34550383
rs12925933
rs76666449
rs117512405
rs6054257
rs762679
rs9955360
rs11699829
rs188918174
rs1332941
rs59409453
rs2276182
rs7772289
rs142730696
rs4695407
rs12451892
rs41304832
rs13062095
rs28502153
rs79977579
rs73730598
rs150150565
rs76065543
rs17677991
rs11579626
rs75664430
rs10773176
rs6751209
rs76219171
rs12412214
rs2282764
rs11991877
rs6587577
rs7790856
rs61748181
rs3785074
rs2056726
rs77732866
rs6790988
rs17445108
rs1611236
rs185174247
rs11769630
rs10112752
rs611646
rs871134
rs3093888
rs2763979
rs4530278
rs9419958
rs112394943
rs131797
rs73581419
rs450962
rs6536702
rs17803849
rs1023767
rs11212631
rs2977608
rs55747751
rs7705526
rs137901416
rs6669563
rs4498805
rs35446936
rs117630647
rs6776756
rs35640778
rs932002
rs141214782
rs143190905
rs56799554
rs41269079
rs11412296
rs66731853
rs13230646
rs10768683
rs939916
rs78491606
rs2967355
rs5742915
rs8105767
rs4743037
rs3891167
rs376641875
rs6659669
rs61405042
rs7221585
rs4724
rs9398196
rs144204502
rs10905255
rs202034370
rs1291143
rs4616688
rs139669835
rs1985369
rs6590343
rs11085072
rs182059586
rs56061761
rs11584821
rs7209057
rs45604339
rs11426156
rs117407747
rs7099229
rs117034449
rs116863223
rs80116508
rs9940099
rs28577594
rs111527438
rs142426306
rs1957937
rs2293579
rs77231040
rs139795227
rs6007020
rs28363070
rs6881568
rs16978028
rs6584579
rs111950327
All − MR Egger
All − Inverse variance weighted
−2
−1
0
1
2
MR effect size for
'telomere length || id:ieu−b−4879' on 'IDP_dMRI_TBSS_L1_Tapetum_L || id:ub
